# Prior infection with SARS-CoV-2 WA1/2020 partially protects rhesus macaques against reinfection with B.1.1.7 and B.1.351 variants

**DOI:** 10.1126/scitranslmed.abj2641

**Published:** 2021-11-03

**Authors:** Abishek Chandrashekar, Jinyan Liu, Jingyou Yu, Katherine McMahan, Lisa H. Tostanoski, Catherine Jacob-Dolan, Noe B. Mercado, Tochi Anioke, Aiquan Chang, Sarah Gardner, Victoria M. Giffin, David L. Hope, Felix Nampanya, Shivani Patel, Owen Sanborn, Daniel Sellers, Huahua Wan, Amanda J. Martinot, John J. Baczenas, Shelby L. O’Connor, Laurent Pessaint, Daniel Valentin, Benjamin Espina, Lauren Wattay, Maria G. Ferrari, Renita Brown, Anthony Cook, Deandre Bueno-Wilkerson, Elyse Teow, Hanne Andersen, Mark G. Lewis, Dan H. Barouch

**Affiliations:** 1 Center for Virology and Vaccine Research, Beth Israel Deaconess Medical Center, Harvard Medical School, Boston, MA 02215, USA.; 2 Harvard Medical School, Boston, MA 02115, USA.; 3 Cummings School of Veterinary Medicine, Tufts University, North Grafton, MA 01536, USA.; 4 Wisconsin National Primate Research Center and University of Wisconsin, Madison, WI 53711, USA.; 5 BIOQUAL, Rockville, MD 20852, USA.; 6 Ragon Institute of MGH, MIT, and Harvard, Cambridge, MA 02139, USA.

## Abstract

Severe acute respiratory syndrome coronavirus 2 (SARS-CoV-2) variants that result in increased transmissibility and partial evasion of neutralizing antibodies have recently emerged. Whether natural immunity induced by the original SARS-CoV-2 WA1/2020 strain protects against rechallenge with these SARS-CoV-2 variants remains a critical unresolved question. In this study, we show that natural immunity induced by the WA1/2020 strain leads to partial but incomplete protection against the SARS-CoV-2 variants B.1.1.7 (alpha) and B.1.351 (beta) in rhesus macaques. We challenged rhesus macaques with B.1.1.7 and B.1.351 and showed that infection with these variants resulted in high viral replication in the upper and lower respiratory tract. We then infected rhesus macaques with the WA1/2020 strain and rechallenged them on day 35 with the WA1/2020, B.1.1.7, or B.1.351 variants. Natural immunity to WA1/2020 led to robust protection against rechallenge with WA1/2020 but only partial protection against rechallenge with B.1.351. An intermediate degree of protection was observed in rhesus macaques against rechallenge with B.1.1.7. These data demonstrate partial but incomplete protective efficacy of natural immunity induced by WA1/2020 against SARS-CoV-2 variants of concern. Our findings have important implications for both vaccination and public health strategies in the context of emerging SARS-CoV-2 variants of concern.

## INTRODUCTION

Severe acute respiratory syndrome coronavirus 2 (SARS-CoV-2) variants (1) have recently emerged, and the proliferation of variants worldwide pose challenges for global pandemic control. B.1.1.7 (alpha) was originally identified in the United Kingdom and has shown increased transmissibility (2, 3), and B.1.351 (beta) was first identified in South Africa and has demonstrated partial evasion of antibody responses (3–7). Previous studies in macaques have demonstrated robust natural immunity against homologous rechallenge with SARS-CoV-2 WA1/2020 (8, 9), but the efficacy of natural immunity induced by the original WA1/2020 strain against SARS-CoV-2 variants of concern has not yet been determined. This is a critical question, because human epidemiologic studies have reported substantial reinfection with SARS-CoV-2 variants in previously infected individuals. For example, studies from Manaus, Brazil demonstrated a surge of coronavirus disease 2019 (COVID-19) infections with the P.1 (gamma) variant in a population with 76% immunity to the original SARS-CoV-2 strain (10, 11). These data suggest that emerging SARS-CoV-2 variants of concern may pose a threat to achieving herd immunity from natural infection. Moreover, SARS-CoV-2 variants may reduce vaccine efficacy. In the present study, we assess the protective efficacy of natural immunity after WA1/2020 infection on rechallenge with WA1/2020, B.1.1.7, or B.1.351 variants in rhesus macaques.

## RESULTS

### Rhesus macaques are susceptible to infection with SARS-CoV-2 B.1.1.7 and B.1.351 variants

We developed SARS-CoV-2 B.1.1.7 and B.1.351 challenge stocks by expansion of seed stocks in Calu-3 cells. Deep sequencing confirmed that the B.1.1.7 and B.1.351 stocks did not contain any unexpected mutations in the spike protein, and specifically, there were no mutations in the furin cleavage site [National Center for Biotechnology Information (NCBI) Sequence Read Archive (SRA) accession numbers SRR14313078 and SRR14313077]. To verify the infectivity of these stocks, we infected six rhesus macaques with 5 × 10^5^ median tissue culture infectious dose (TCID_50_) B.1.1.7 (*n* = 3) or B.1.351 (*n* = 3) by the intranasal and intratracheal routes. Both viruses resulted in viral replication in the upper and lower respiratory tracts, as measured by subgenomic RNA (sgRNA) (12, 13). Median peak log sgRNA copies per milliliter in bronchoalveolar lavage (BAL) were 5.59 and 6.05, and peak log sgRNA copies per swab in nasal swabs were 5.61 and 5.60 for the B.1.1.7- and B.1.351-infected animals, respectively (figs. S1 and S2). Viral replication was observed for about 10 days, although several animals still had detectable sgRNA on day 10. Animals infected with B.1.1.7 and B.1.351 did not develop appreciable clinical disease. Using a luciferase-based pseudovirus neutralizing antibody (NAb) assay (14), we observed that macaques infected with the B.1.1.7 variant generated comparable NAb titers to the WA1/2020, D614G, and B.1.1.7 strains but lower NAb titers to the B.1.351 variant, whereas infection with B.1.351 induced higher NAb titers to B.1.351 than these other strains (fig. S3).

### Rechallenge of SARS-CoV-2 WA1/2020–infected macaques with B.1.1.7 or B.1.351 variants results in incomplete protection

**Fig. 1.**
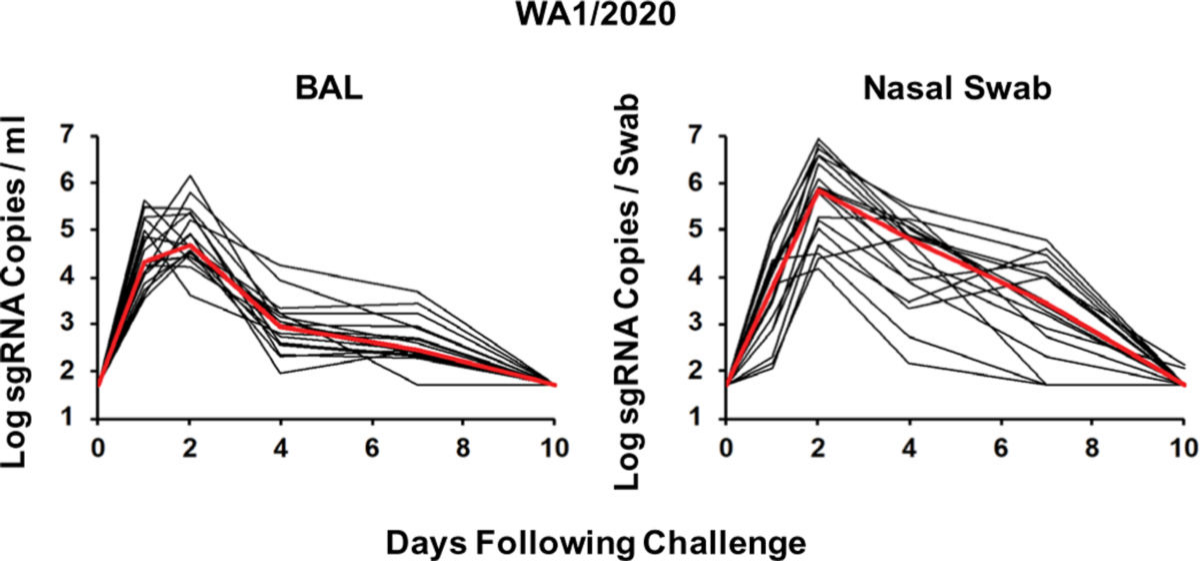
Rhesus macaques are susceptible to SARS-CoV-2 WA1/2020 infection. Rhesus macaques were infected by the intranasal and intratracheal routes with 5×10^5^ TCID_50_ SARS-CoV-2 WA1/2020 (*n* = 18). Peak log_10_ sgRNA copies per milliliter (limit of quantification 50 copies/ml) were assessed in BAL after challenge, and peak log_10_ sgRNA copies per swab (limit of quantification 50 copies per swab) were assessed in nasal swabs after challenge. Red lines reflect median values.

Most people who were infected with SARS-CoV-2 in 2020 were infected with the original Wuhan or WA1/2020 strain or the D614G variant (1). The B.1.1.7 variant (2) and the B.1.351 variant (6, 7) have now spread globally. The B.1.351 variant is particularly worrisome for natural and vaccine immunity, because it contains mutations in the receptor binding domain (RBD), including E484K, and the N-terminal domain (NTD), which collectively lead to partial escape from NAb responses (3–7). To assess the extent of protective efficacy of WA1/2020 natural immunity against the B.1.1.7 and B.1.351 variants, we infected 18 rhesus macaques with 5 × 10^5^ TCID_50_ WA1/2020 by the intranasal and intratracheal routes. All animals developed high viral replication in BAL and nasal swabs, with peak log sgRNA copies per milliliter in BAL of 4.69 (range 3.60 to 6.15) and peak log sgRNA copies per swab in nasal swabs of 5.86 (range 4.17 to 6.95) on day 2 after infection (Fig. 1), similar to our prior reports (8, 9, 15, 16). Viral replication resolved by day 10 in the majority of animals.

**Fig. 2.**
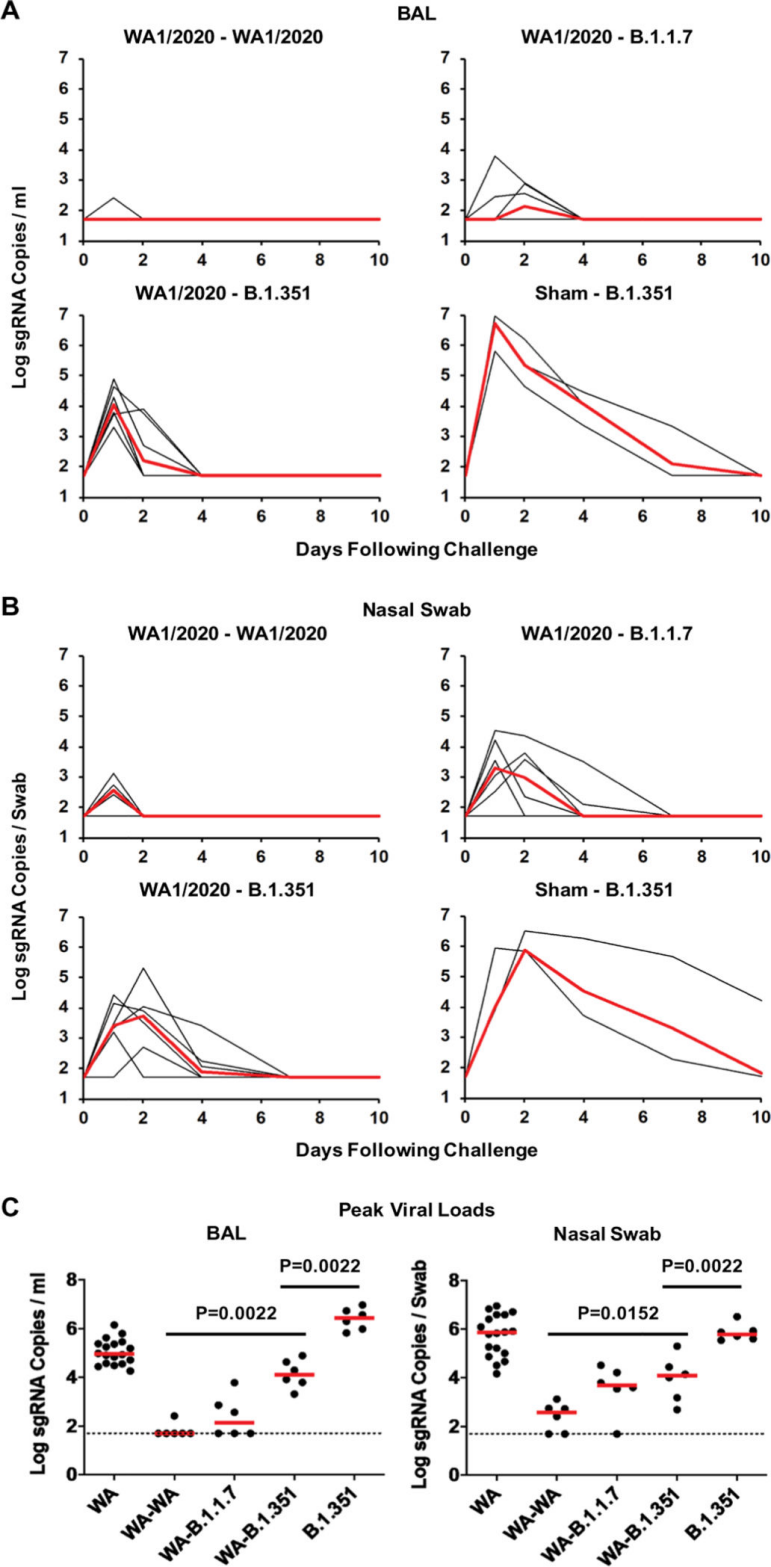
Prior infection with SARS-CoV-2 WA1/2020 protects against rechallenge with WA1/2020, B.1.1.7, or B.1.351 variants. SARS-CoV-2 WA1/2020–infected rhesus macaques were rechallenged on day 35 by the intranasal and intratracheal routes with 5 × 10^5^ TCID_50_ SARS-CoV-2 WA1/2020, B.1.1.7, or B.1.351 (*n* = 6 per group). Naïve animals (*n* = 3) were also infected with B.1.351 as a concurrent positive control. (**A**) Peak log_10_ sgRNA copies per milliliter (limit of quantification 50 copies/ml) were assessed in BAL after challenge. (**B**) Peak log_10_ sgRNA copies per swab (limit of quantification 50 copies per swab) were assessed in nasal swabs after challenge. Red lines reflect median values. (**C**) Peak viral loads in BAL and nasal swabs after challenge are shown. Horizontal red bars reflect median values. *P* values reflect two-sided Wilcoxon rank sum tests. WA reflects naïve animals after initial infection with WA1/2020; WA-WA, WA-B.1.1.7, and WA-B.1.351 reflect WA1/2020 rechallenged with WA1/2020, B.1.1.7, and B.1.351, respectively. The B.1.351-infected animals in this panel include the three animals from the initial infection experiment (fig. S2) as well as the three animals that were concurrently infected in this experiment.

On day 35 after initial infection, we rechallenged these 18 animals with 5 × 10^5^ TCID_50_ WA1/2020, B.1.1.7, or B.1.351 by the intranasal and intratracheal routes (*n* = 6 per group) (fig. S4). An additional three naïve macaques were concurrently challenged with 5 × 10^5^ TCID_50_ B.1.351 as positive controls. Rechallenge of WA1/2020 convalescent animals with WA1/2020 resulted in undetectable sgRNA copies per milliliter in BAL, except for a single animal that showed a low blip of detectable virus on day 1, and a median peak of 2.57 (range of less than 1.70 to 3.12) log sgRNA copies per swab in nasal swabs (Fig. 2, A and B). Animals rechallenged with B.1.1.7 developed slightly higher concentrations of breakthrough virus, including a median peak of 2.13 (range of less than 1.70 to 2.90) log sgRNA copies per milliliter in BAL and a median peak of 3.30 (range of less than 1.70 to 4.52) log sgRNA copies per swab in nasal swabs. In contrast, animals rechallenged with B.1.351 developed higher concentrations of breakthrough virus, including a median peak of 4.04 (range 3.31 to 4.89) log sgRNA copies per milliliter in BAL and a median peak of 3.71 (range of less than 1.70 to 5.30) log sgRNA copies per swab in nasal swabs. Deep sequencing of breakthrough B.1.351 virus in BAL after rechallenge showed no mutations compared with the B.1.351 viral stock sequence (NCBI SRA BioProject PRJNA744242). Naïve animals that were concurrently infected with B.1.351 demonstrated a median peak of 6.73 (range 5.82 to 6.97) log sgRNA copies per milliliter in BAL and a median peak of 5.87 (range 5.84 to 6.51) log sgRNA copies per swab in nasal swabs, consistent with the initial infection study (fig. S2).

In WA1/2020-infected animals that were rechallenged, median peak viral loads were greater than 2.38 and 1.51 logs higher after rechallenge with B.1.351 than rechallenge with WA1/2020 in BAL and nasal swabs, respectively (*P* = 0.0022 and *P* = 0.0152, respectively, two-sided Wilcoxon rank sum tests; Fig. 2C). Moreover, median peak viral loads in previously naïve animals that were infected with B.1.351 were 2.35 and 1.71 logs higher than in WA1/2020 convalescent macaques that were rechallenged with B.1.351 in BAL and nasal swabs, respectively (*P* = 0.0022 and *P* = 0.0022, respectively, two-sided Wilcoxon rank sum tests; Fig. 2C). Together, these data demonstrate that natural immunity to WA1/2020 provided partial but incomplete protection against rechallenge with B.1.351 in macaques. Intermediate outcomes were observed after rechallenge with B.1.1.7. Median peak viral loads after B.1.351 infection of naïve macaques were 1.48 logs higher than after WA1/2020 infection of naïve macaques in BAL (Figs. 1 and 2, A and C, and fig. S2), suggesting increased viral replicative capacity of B.1.351 compared with WA1/2020 in the lower respiratory tract in this model, although viral loads in nasal swabs appeared comparable (Figs. 1 and 2, A and C, and fig. S2).

### Rechallenge with SARS-CoV-2 variants increased NAb responses

**Fig. 3.**
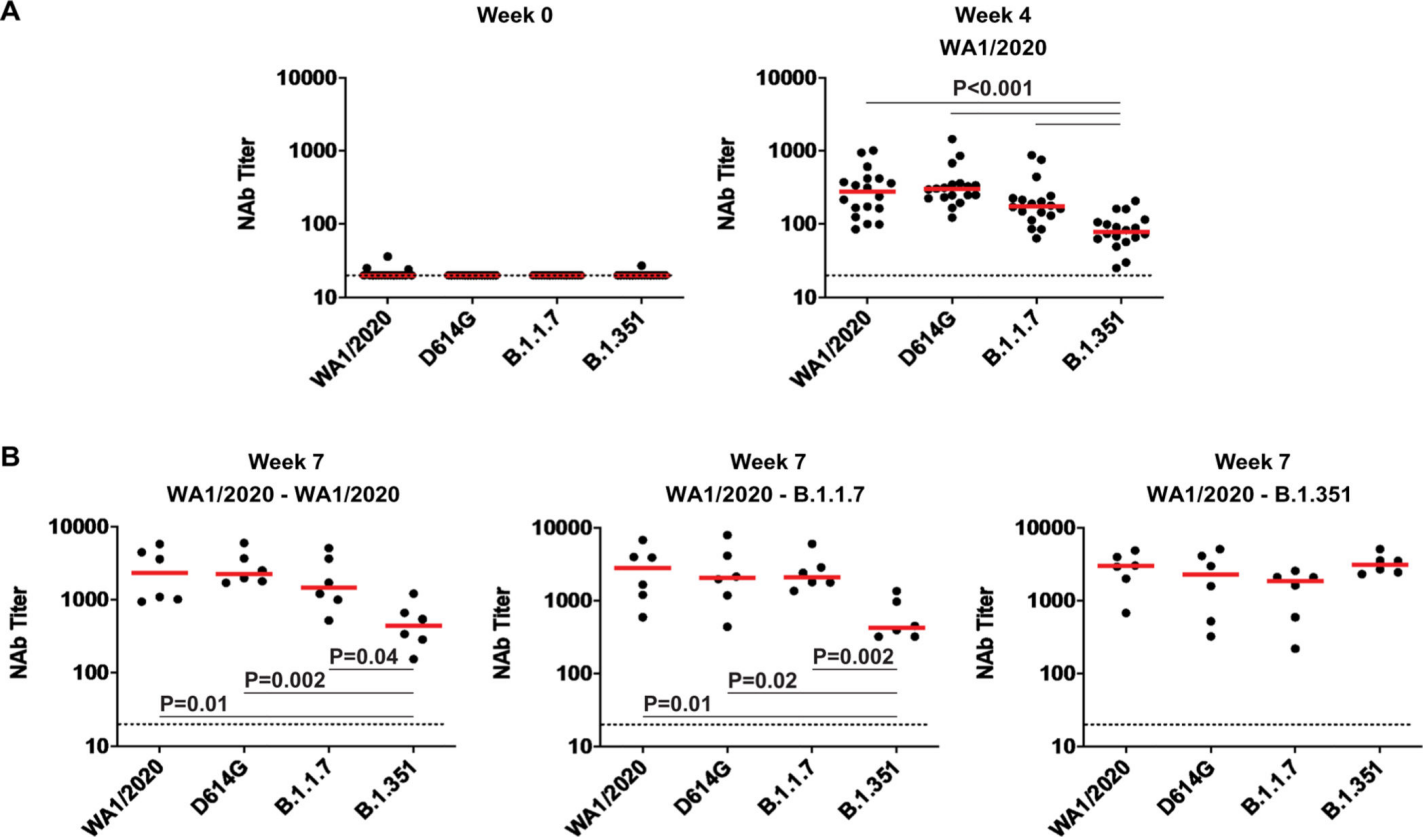
NAb responses are observed against variants in previously infected rhesus macaques. (**A**) Pseudovirus NAb assays against the SARS-CoV-2 WA1/2020, D614G, B.1.1.7, and B.1.351 variants were assessed at weeks 0 and 4 after primary WA1/2020 infection. (**B**) Pseudovirus Nab assays are shown for samples collected at week 7 after rechallenge with WA1/2020, B.1.1.7, and B.1.351. Horizontal red bars reflect median responses. Dotted lines reflect assay limit of quantitation. *P* values reflect two-sided Wilcoxon rank sum tests.

**Fig. 4.**
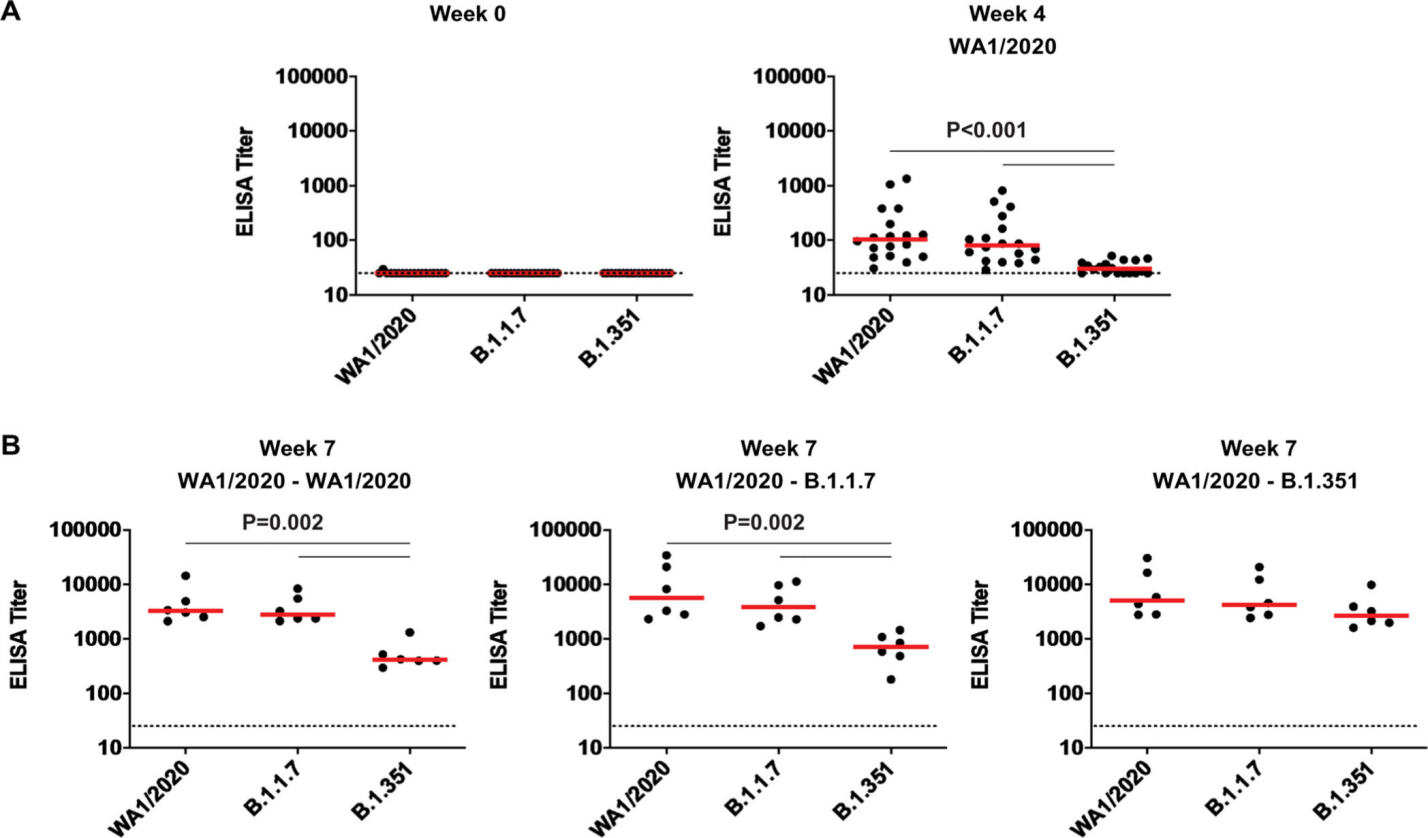
Binding antibody responses against B.1.1.7 and B.1.351 are lower than responses against WA1/2020 after rechallenge of rhesus macaques. (**A** and **B**) RBD-specific binding antibody responses against the SARS-CoV-2 WA1/2020, B.1.1.7, and B.1.351 variants were assessed at weeks 0 and 4 after primary WA1/2020 infection (A) and at week 7 after rechallenge with WA1/2020, B.1.1.7, and B.1.351 (B). Horizontal red bars reflect median responses. Dotted lines reflect assay limit of quantitation. *P* values reflect two-sided Wilcoxon rank sum tests.

At week 4 after primary WA1/2020 infection, macaques developed median NAb titers of 277, 302, 175, and 78 against WA1/2020, D614G, B.1.1.7, and B.1.351 viruses, respectively (Fig. 3A). Rechallenge with WA1/2020 or B.1.1.7 led to increased NAb responses to all variants, with the lowest responses to B.1.351, whereas rechallenge with B.1.351 led to comparable NAb titers to all variants tested (Fig. 3B). Binding antibodies were assessed by an RBD enzyme-linked immunosorbent assay (ELISA) and showed similar patterns. No RBD-specific antibody responses were observed before infection (Fig. 4A). Infection with WA1/2020 and rechallenge with WA1/2020 or B.1.1.7 resulted in higher responses against WA1/2020 and B.1.1.7 but lower responses to B.1.351, whereas rechallenge with B.1.351 led to comparable responses against all variants tested (Fig. 4B). Similarly, an electrochemiluminescence assay (ECLA) (17) showed that rechallenge with WA1/2020 and B.1.1.7 led to lower responses to B.1.351 and P.1 RBD compared with WA1/2020 and B.1.1.7 RBD, although responses to full-length spike were comparable (fig. S5). These data suggest that variant-specific antibody responses were directed more against RBD than full-length spike protein.

To assess potential immune correlates of protection, we compared humoral immune responses before rechallenge with viral loads after rechallenge. ELISA and NAb titers against the rechallenge virus before rechallenge correlated with peak sgRNA copies per milliliter in BAL after rechallenge (*P* < 0.0001, *R* = −0.8302 and *P* = 0.0007, *R* = −0.7243, respectively; fig. S6). Similarly, NAb titers before rechallenge also correlated with peak sgRNA copies per swab in nasal swabs (*P* = 0.0150, *R* = −0.5631; fig. S7) after rechallenge.

**Fig. 5.**
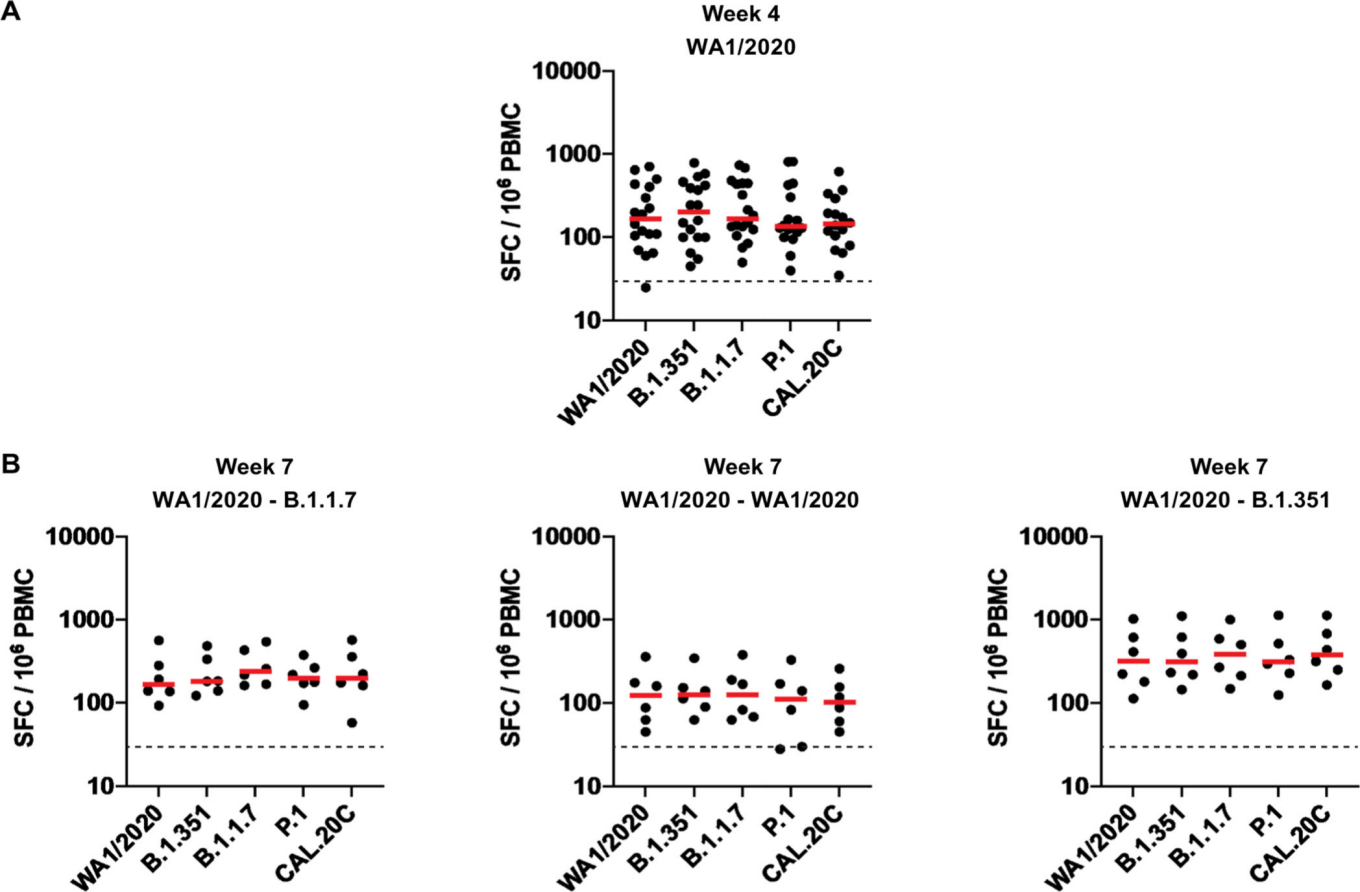
T cell responses are comparable in infected rhesus macaques after rechallenge with WA1/2020, B.1.1.7, and B.1.351. (**A** and **B**) Cellular immune responses to pooled spike protein peptides were assessed by IFN-γ ELISPOT assays to WA1/2020, B.1.351, B.1.1.7, P.1, and CAL.20C variants at week 4 after primary WA1/2020 infection (A) and at week 7 after rechallenge with WA1/2020, B.1.1.7, and B.1.351 (B). Horizontal red bars reflect median responses. Dotted lines reflect assay limit of quantitation. SFC, spot-forming cells.

In contrast with the differences observed with antibody responses against certain variants, T cell responses after infection with WA1/2020 and after rechallenge with WA1/2020, B.1.1.7, and B.1.351 were comparable across all variants tested. Spike protein–specific cellular immune responses were assessed by pooled peptide interferon (IFN)–γ enzyme-linked immunospot (ELISPOT) and intracellular cytokine staining (ICS) assays using peripheral blood mononuclear cells (PBMCs). Cellular immune responses were comparable by IFN-γ ELISPOT assays against multiple SARS-CoV-2 variants, including WA1/2020, B.1.351, B.1.1.7, P.1, and CAL.20C (Fig. 5). Similarly, multiparameter ICS assays demonstrated comparable responses for total and CD28^+^CD95^+^ central memory CD8^+^ and CD4^+^ T cells against these variants (figs. S8 and S9).

### Rhesus macaques infected with B.1.1.7 and B.1.351 strains of SARS-CoV-2 were protected against reinfection with the WA1/2020 strain

In a pilot experiment, we rechallenged the six macaques originally infected with B.1.1.7 and B.1.351 (figs. S1 and S2) with WA1/2020 on day 35 after initial infection. Macaques initially infected with B.1.1.7 demonstrated no detectable viral loads in BAL or nasal swabs after rechallenge with WA1/2020 (figs. S10 and S11). In contrast, one of the three macaques initially infected with B.1.351 showed breakthrough virus in BAL and nasal swabs after rechallenge with WA1/2020 (figs. S10 and S11). These data further demonstrate partial protection after rechallenge with heterologous SARS-CoV-2 variants.

## DISCUSSION

Multiple SARS-CoV-2 variants have recently emerged, some of which increase transmissibility and pathogenicity, and some of which partially evade NAbs induced by the Wuhan, WA1/2020, and D614G strains (1–7). Our data demonstrate that natural immunity induced by the WA1/2020 strain provides partial but incomplete protection against the B.1.351 variant in rhesus macaques. These data are consistent with human epidemiologic studies that have documented COVID-19 surges with partially resistant SARS-CoV-2 variants in populations with a high degree of immunity to the original strain (10, 11). Together, these findings demonstrate that natural immunity provides robust short-term protection against rechallenge with the same SARS-CoV-2 strain but reduced protection against rechallenge with certain SARS-CoV-2 variants.

These data build on prior studies from our laboratory and others, showing that infection of macaques with the WA1/2020 strain led to robust natural immunity against rechallenge with the same strain (8, 18, 19). Our current data confirm and extend these studies and define the protective efficacy of natural immunity induced by WA1/2020 against the B.1.1.7 and B.1.351 SARS-CoV-2 variants of concern in this model. Further work will be required to define mechanistic correlates of protection against SARS-CoV-2 variants. In particular, whether the partial protection of WA1/2020 natural immunity against B.1.351 resulted from the reduced but still detectable antibody responses or the robust and unchanged T cell responses, or a combination of these immune parameters, remains to be determined.

We previously reported that purified immunoglobulin G (IgG) was sufficient to protect macaques against SARS-CoV-2 if antibody titers exceeded a threshold, but that CD8^+^ T cells also contributed to protection if antibodies were subprotective (9). Robust CD4^+^ and CD8^+^ T cell responses have been reported in SARS-CoV-2–infected humans by our laboratory and others (20–23). The potential role of CD8^+^ and CD4^+^ T cells in natural or vaccine protection against SARS-CoV-2 variants remains to be determined, but a contribution of cellular immune responses in protecting against SARS-CoV-2 variants that partially evade antibody responses may be plausible. In the present study, the reduction of NAb titers to B.1.351 compared with WA1/2020, with unchanged T cell responses, likely accounts for the observed reduced protection.

Our data also demonstrate that B.1.351 infection led to more rapid kinetics, higher magnitude, and more prolonged virus replication than did WA1/2020 infection in rhesus macaques. If B.1.351 or other variants similarly lead to more robust virus replication in humans, then such variants would likely be more transmissible and less well controlled by vaccines, with substantial implications for public health. Further research will be required to compare viral loads with additional viral variants in nonhuman primates and humans.

Our study has several limitations. First, studies of viral variants in nonhuman primates may not be fully translatable to humans, because the virus specifically adapts to humans. Second, our study only assessed the B.1.1.7 (alpha) and B.1.351 (beta) variants, and thus further studies will be required to evaluate other variants, such as the B.1.617.2 (delta) variant. Third, our data do not evaluate the durability of immune responses associated with protection against viral variants.

Together, our data suggest partial but reduced short-term protective efficacy of WA1/2020 natural immunity against SARS-CoV-2 variants of concern. A greater reduction of efficacy was observed against B.1.351 as compared with B.1.1.7, likely because B.1.351 includes multiple RBD and NTD mutations that partially evade NAb responses. Our findings have important implications for vaccination and public health strategies in regions that have circulating SARS-CoV-2 variants of concern. Moreover, future studies will define protective efficacy against other SARS-CoV-2 variants and will define immunologic mechanisms of protection against these variants.

## MATERIALS AND METHODS

### Study design

Twenty-seven outbred Indian origin adult male and female rhesus macaques (*Macaca mulatta*) ages 6 to 10 years old were randomly allocated to groups. All animals were housed at BIOQUAL Inc. Animals were challenged with 5 × 10^10^ TCID_50_ WA1/2020, B.1.1.7, or B.1.351 and then rechallenged with WA1/2020, B.1.1.7, or B.1.351 on day 35. The WA1/2020 (USA-WA1/2020; BEI Resources, NR-5228) challenge stock was described previously (8). The B.1.1.7 (USA/CA_CDC_5574/2020; BEI Resources, NR-54011) and B.1.351 (South Africa/KRISP-K005325/2020; BEI Resources, NR-54974) challenge stocks were grown in Calu-3 cells (American Type Culture Collection HTB-55). Deep sequencing of these stocks revealed no unexpected mutations in the spike protein at any position at greater than 2.5% frequency. Moreover, the B.1.351 stock contained no unexpected mutations elsewhere in virus at >13% frequency. All the sequence data generated from viruses isolated from animals can be found in NCBI SRA BioProject PRJNA744242. The sequence of the challenge stock can be found under the NCBI SRA accession numbers SRR14313078 and SRR14313077. Virus was administered as 1 ml by the intranasal route (0.5 ml in each nare) and 1 ml by the intratracheal route. All immunologic and virologic studies were performed blinded. Animal studies were conducted in compliance with all relevant local, state, and federal regulations and were approved by the BIOQUAL Institutional Animal Care and Use Committee.

### Pseudovirus-based virus neutralization assay

The SARS-CoV-2 pseudoviruses expressing a luciferase reporter gene were generated essentially as described previously (8, 14–16). Briefly, the packaging plasmid psPAX2 (AIDS Resource and Reagent Program), luciferase reporter plasmid pLenti-CMV Puro-Luc (Addgene), and spike protein expressing pcDNA3.1-SARS CoV-2 SΔCT of variants were cotransfected into human embryonic kidney (HEK) 293T cells by Lipofectamine 2000 (Thermo Fisher Scientific). Pseudoviruses of SARS-CoV-2 variants were generated by using WA1/2020 strain [Wuhan/WIV04/2019, GISAID accession identification (ID): EPI_ISL_402124], D614G mutation, B.1.1.7 variant (GISAID accession ID: EPI_ISL_601443), or B.1.351 variant (GISAID accession ID: EPI_ISL_712096). The supernatants containing the pseudotype viruses were collected 48 hours post transfection, which were purified by centrifugation and filtration with a 0.45-μm filter. To determine the neutralization activity of the plasma or serum samples from participants, HEK293T–human angiotensin converting enzyme 2 (hACE2) cells (8, 14–16) were seeded in 96-well tissue culture plates at a density of 1.75 × 10^4^ cells per well overnight. Threefold serial dilutions of heat-inactivated serum or plasma samples were prepared and mixed with 50 μl of pseudovirus. The mixture was incubated at 37°C for 1 hour before adding to HEK293T-hACE2 cells. Forty-eight hours after infection, cells were lysed in Steady-Glo Luciferase Assay (Promega) according to the manufacturer’s instructions. SARS-CoV-2 neutralization titers were defined as the sample dilution at which a 50% reduction in relative light unit (RLU) was observed relative to the average of the virus control wells.

### Enzyme-linked immunosorbent assay

WA1/2020, B.1.1.7, and B.1.351 RBD-specific binding antibodies were assessed by ELISA essentially as described previously (8, 15, 16). Briefly, 96-well plates were coated with RBD protein (0.5 μg/ml) in 1× Dulbecco’s phosphate-buffered saline (DPBS) and incubated at 4°C overnight. After incubation, plates were washed once with wash buffer (0.05% Tween 20 in 1× DPBS) and blocked with 350 μl of casein block (Thermo Fisher Scientific) per well for 2 to 3 hours at room temperature. After incubation, block solution was discarded, and plates were blotted dry. Serial dilutions of heat-inactivated serum diluted in casein block were added to wells, and plates were incubated for 1 hour at room temperature before three further washes and a 1-hour incubation with a 1 μg/ml dilution of anti-macaque IgG horseradish peroxidase (Nonhuman Primate Reagent Resource) at room temperature in the dark. Plates were then washed three times, and 100 μl of SeraCare KPL 3,3′,5,5′-tetramethylbenzidine (TMB) SureBlue Start solution was added to each well; plate development was halted by the addition of 100 μl of SeraCare KPL TMB Stop solution per well. The absorbance at 450 nm was recorded using a VersaMax microplate reader. For each sample, ELISA end point titer was calculated in GraphPad Prism software using a four-parameter logistic curve fit to calculate the reciprocal serum dilution that yields an absorbance value of 0.2 at 450 nm. Log_10_ end point titers are reported.

### Electrochemiluminescence assay

ECLA plates (Meso Scale Discovery SARS-CoV-2 IgG cat. no.: N05CA-1; Panel 7) were designed and produced with up to nine antigen spots in each well, and assays were performed essentially as described previously (17). The antigens included were WA1/2020, B.1.1.7, P.1, and B.1.351 spike protein and RBD. The plates were blocked with 50 μl of Blocker A (1% bovine serum albumin in Milli-Q water) solution for at least 30 min at room temperature shaking at 700 rpm with a digital microplate shaker. During blocking, the serum was diluted 1:5000 in Diluent 100. The plates were then washed three times with 150 μl of the Meso Scale Discovery (MSD) kit wash buffer and blotted dry, and 50 μl of the diluted samples was added in duplicate to the plates and set to shake at 700 rpm at room temperature for at least 2 hours. The plates were again washed three times, and 50 μl of SULFO-tagged anti-human IgG detection antibody diluted to 1× in Diluent 100 was added to each well and incubated shaking at 700 rpm at room temperature for at least 1 hour. Plates were then washed three times, 150 μl of MSD GOLD Read buffer B was added to each well, and the plates were read immediately after on a MESO QuickPlex SQ 120 machine. MSD titers for each sample were reported as RLUs, which were calculated as sample RLU minus blank RLU for each spot for each sample. The limit of detection was defined as 1000 RLU for each assay.

### IFN-γ ELISPOT assay

ELISPOT assays were performed using PBMCs essentially as described previously (8, 15, 16). Peptide pools consisted of 15 amino acid peptides overlapping by 11 amino acids spanning the SARS-CoV-2 spike protein from the WA1/2020 strain or variant strains. ELISPOT plates were coated with mouse anti-human IFN-γ mono-clonal antibody (mAb) from BD Pharmingen at 5 μg per well and incubated overnight at 4°C. Plates were washed with DPBS wash buffer (DPBS with 0.25% Tween 20) and blocked with R10 media [RPMI with 10% heat-inactivated fetal bovine serum (FBS) with 1% of 100× penicillin-streptomycin] for 1 to 4 hours at 37°C. SARS-CoV-2 peptides (21st Century Biochemicals; the variants peptides contain the wild-type backbone) were prepared and plated at a concentration of 1 μg per well, and 200,000 cells per well were added to the plate. The peptides and cells were incubated for 18 to 24 hours at 37°C. Positive control wells were cells with phytohemagglutinin, and negative control wells were cells with media. All steps following this incubation were performed at room temperature. The plates were washed with ELISPOT wash buffer (11% 10× DPBS and 0.3% Tween20 in 1 liter of Milli-Q water) and incubated for 2 hours with rabbit polyclonal anti-human IFN-γ biotin from U-CyTech (1 μg/ml). The plates were washed a second time and incubated for 2 hours with streptavidin-alkaline phosphatase from SouthernBiotech (2 μg/ml). The final wash was followed by the addition of nitro-blue tetrazolium chloride/5-bromo-4-chloro 3′-indolyphosphate p-toluidine salt (chromagen) substrate solution for 7 min. The chromagen was discarded, and the plates were washed with water and dried in a dim place for 24 hours. Plates were scanned and counted on a Cellular Technology Limited immunospot analyzer.

### ICS assay

Multiparameter ICS assays were performed essentially as described previously (8, 15, 16). PBMCs (10^6^) per well were resuspended in 100 μl of R10 media [RPMI 1640 (Thermo Fisher Scientific) with 10% FBS (Omega) and 1% of 100× penicillin-streptomycin (Thermo Fisher Scientific)] supplemented with CD49d mAb (1 μg/ml). Each sample was assessed with mock (100 μl of R10 plus 0.5% dimethyl sulfoxide; background control), SARS-CoV-2 peptides (2 μg/ml; 21st Century Biochemicals; the variants peptides contain the wild-type backbone), or phorbol myristate acetate (10 pg/ml) and ionomycin (1 μg/ml; Sigma-Aldrich) (100 μl; positive control) and incubated at 37°C for 1 hour. After incubation, 0.25 μl of GolgiStop (BD Biosciences) and 0.25 μl of GolgiPlug (BD Biosciences) in 50 μl of R10 was added to each well and incubated at 37°C for 8 hours and then held at 4°C overnight. The next day, the cells were washed twice with DPBS, stained with Near-Infrared Fixable LIVE/DEAD dye (Life Technologies; diluted in DPBS 1:100 and 10 μl per well) for 10 min, and then stained with mAbs against CD279 (clone EH12.1, Brilliant Blue 700, 2 μl per well; BD Pharmingen), CD38 [clone OKT10, Phycoerythrin (PE), 0.5 μl per well; NHP Reagent Resource], CD28 (clone 28.2, PE-cyanine 5, 2.5 μl per well; BD Pharmingen), CD4 (clone L200, Brilliant Violet 510, 0.625 μl per well; BD Pharmingen), CD95 [clone DX2, Brilliant Ultra Violet (BUV) 737, 0.5 μl per well; BD Pharmingen], and CD8 (clone SK1, BUV 805, 1 μl per well; BD Pharmingen) for 30 min. Cells were then washed twice with 2% FBS in DPBS buffer and incubated for 15 min with 200 μl of BD CytoFix/CytoPerm Fixation/Permeabilization solution. Cells were washed twice with 1× Perm Wash buffer (BD Perm/Wash buffer 10× in the CytoFix/CytoPerm Fixation/Permeabilization kit diluted with Milli-Q water and passed through a 0.22-μm filter) and stained intracellularly with mAbs against CD69 (clone TP1.55.3, energy-coupled dye/PE-Texas Red, 0.625 μl per well; Beckman Coulter), IFN-γ (clone B27; BUV 395, 2.5 μl per well; BD Pharmingen), CD45 (clone D058–1283, BUV 615, 0.05 μl per well; BD Pharmingen), and CD3 (clone SP34.2, Alexa Fluor 700, 0.25 μl per well; BD Pharmingen) for 30 min. Cells were washed twice with 1× Perm Wash buffer and fixed with 250 μl of freshly prepared 1.5% formaldehyde. Fixed cells were transferred to a 96-well round-bottom plate and analyzed by BD FACSymphony system.

### sgRNA assay

SARS-CoV-2 E gene sgRNA was assessed by reverse transcription polymerase chain reactions (RT-PCR) using primers and probes as previously described (12, 13). A standard was generated by first synthesizing a gene fragment of the subgenomic E gene (12). The gene fragment was subsequently cloned into a pcDNA3.1^+^ expression plasmid using restriction site cloning (Integrated DNA Technologies). The insert was in vitro transcribed to RNA using the AmpliCap-Max T7 High Yield Message Maker Kit (CELLSCRIPT). Log dilutions of the standard were prepared for RT-PCR assays ranging from 1 × 10^10^ copies to 1 × 10^−1^ copies. Viral loads were quantified from BAL fluid and nasal swabs. RNA extraction was performed on a QIAcube HT using the IndiSpin QIAcube HT Pathogen Kit according to the manufacturer’s specifications (QIAGEN). The standard dilutions and extracted RNA samples were reverse-transcribed using SuperScript VILO Master Mix (Invitrogen) following the cycling conditions described by the manufacturer, 25°C for 10 min, 42°C for 1 hour, and then 85°C for 5 min. A TaqMan custom gene expression assay (Thermo Fisher Scientific) was designed using the sequences targeting the E gene sgRNA (12). The sequences for the custom assay were as follows: forward primer, sgLeadCoV2.Fwd: CGATCTCTTGTAGATCTGTTCTC; E_Sarbeco_R: ATATTGCAG-CAGTACGCACACA; and E_Sarbeco_P1 (probe): VIC-ACACTAG-CCATCCTTACTGCGCTTCG-MGB. These primers and probes were equally reactive for both variants. Reactions were carried out in duplicate for samples and standards on the QuantStudio 6 and 7 Flex Real-Time PCR Systems (Applied Biosystems) with the thermal cycling conditions, initial denaturation at 95°C for 20 s, then 45 cycles of 95°C for 1 s, and 60°C for 20 s. Standard curves were used to calculate sgRNA copies per milliliter or per swab; the quantitative assay sensitivity was 50 copies per milliliter or per swab.

### Statistical analyses

Comparisons of virologic and immunologic data were performed using GraphPad Prism 8.4.2 (GraphPad Software). Comparison of data between groups was performed using two-sided Wilcoxon rank sum tests. Correlation analyses were performed using two-sided Spearman rank correlation tests. *P* values of less than 0.05 were considered significant.

## Supplementary Material

Data file S1

Supplemental Figures

## References

[1] Korber B, Fischer WM, Gnanakaran S, Yoon H, Theiler J, Abfalterer W, Hengartner N, Giorgi EE, Bhattacharya T, Foley B, Hastie KM, Parker MD, Partridge DG, Evans CM, Freeman TM, de Silva TI, Tracking changes in SARS-CoV-2 Spike: Evidence that D614G increases infectivity of the COVID-19 virus. Cell 182, 812–827.e19 (2020). 32697968 10.1016/j.cell.2020.06.043 PMC7332439

[2] Davies NG, Abbott S, Barnard RC, Jarvis CI, Kucharski AJ, Munday JD, Pearson CAB, Russell TW, Tully DC, Washburne AD, Wenseleers T, Gimma A, Waites W, Wong KLM, van Zandvoort K, Silverman JD; CMMID COVID-Working Group; COVID-Genomics UK (COG-UK) Consortium, Diaz-Ordaz K, Keogh R, Eggo RM, Funk S, Jit M, Atkins KE, Edmunds WJ, Estimated transmissibility and impact of SARS-CoV-2 lineage B.1.1.7 in England. Science 372, eabg3055 (2021). 33658326 10.1126/science.abg3055 PMC8128288

[3] Wang P, Nair MS, Liu L, Iketani S, Luo Y, Guo Y, Wang M, Yu J, Zhang B, Kwong PD, Graham BS, Mascola JR, Chang JY, Yin MT, Sobieszczyk M, Kyratsous CA, Shapiro L, Sheng Z, Huang Y, Ho DD, Antibody resistance of SARS-CoV-2 variants B.1.351 and B.1.1.7. Nature 593, 130–135 (2021). 33684923 10.1038/s41586-021-03398-2

[4] Wu K, Werner AP, Koch M, Choi A, Narayanan E, Stewart-Jones GBE, Colpitts T, Bennett H, Boyoglu-Barnum S, Shi W, Moliva JI, Sullivan NJ, Graham BS, Carfi A, Corbett KS, Seder RA, Edwards DK, Serum neutralizing activity elicited by mRNA-1273 vaccine. N. Engl. J. Med 384, 1468–1470 (2021). 33730471 10.1056/NEJMc2102179 PMC8008744

[5] Liu Y, Liu J, Xia H, Zhang X, Fontes-Garfias CR, Swanson KA, Cai H, Sarkar R, Chen W, Cutler M, Cooper D, Weaver SC, Muik A, Sahin U, Jansen KU, Xie X, Dormitzer PR, Shi PY, Neutralizing activity of BNT162b2-elicited serum. N. Engl. J. Med 384, 1466–1468 (2021). 33684280 10.1056/NEJMc2102017 PMC7944950

[6] Wibmer CK, Ayres F, Hermanus T, Madzivhandila M, Kgagudi P, Oosthuysen B, Lambson BE, de Oliveira T, Vermeulen M, van der Berg K, Rossouw T, Boswell M, Ueckermann V, Meiring S, von Gottberg A, Cohen C, Morris L, Bhiman JN, Moore PL, SARS-CoV-2 501Y.V2 escapes neutralization by South African COVID-19 donor plasma. Nat. Med 27, 622–625 (2021). 33654292 10.1038/s41591-021-01285-x

[7] Tegally H, Wilkinson E, Giovanetti M, Iranzadeh A, Fonseca V, Giandhari J, Doolabh D, Pillay S, San EJ, Msomi N, Mlisana K, von Gottberg A, Walaza S, Allam M, Ismail A, Mohale T, Glass AJ, Engelbrecht S, van Zyl G, Preiser W, Petruccione F, Sigal A, Hardie D, Marais G, Hsiao NY, Korsman S, Davies MA, Tyers L, Mudau I, York D, Maslo C, Goedhals D, Abrahams S, Laguda-Akingba O, Alisoltani-Dehkordi A, Godzik A, Wibmer CK, Sewell BT, Lourenço J, Alcantara LCJ, Kosakovsky Pond SL, Weaver S, Martin D, Lessells RJ, Bhiman JN, Williamson C, de Oliveira T, Detection of a SARS-CoV-2 variant of concern in South Africa. Nature 592, 438–443 (2021). 33690265 10.1038/s41586-021-03402-9

[8] Chandrashekar A, Liu J, Martinot AJ, McMahan K, Mercado NB, Peter L, Tostanoski LH, Yu J, Maliga Z, Nekorchuk M, Busman-Sahay K, Terry M, Wrijil LM, Ducat S, Martinez DR, Atyeo C, Fischinger S, Burke JS, Slein MD, Pessaint L, van Ry A, Greenhouse J, Taylor T, Blade K, Cook A, Finneyfrock B, Brown R, Teow E, Velasco J, Zahn R, Wegmann F, Abbink P, Bondzie EA, Dagotto G, Gebre MS, He X, Jacob-Dolan C, Kordana N, Li Z, Lifton MA, Mahrokhian SH, Maxfield LF, Nityanandam R, Nkolola JP, Schmidt AG, Miller AD, Baric RS, Alter G, Sorger PK, Estes JD, Andersen H, Lewis MG, Barouch DH, SARS-CoV-2 infection protects against rechallenge in rhesus macaques. Science 369, 812–817 (2020). 32434946 10.1126/science.abc4776 PMC7243369

[9] McMahan K, Yu J, Mercado NB, Loos C, Tostanoski LH, Chandrashekar A, Liu J, Peter L, Atyeo C, Zhu A, Bondzie EA, Dagotto G, Gebre MS, Jacob-Dolan C, Li Z, Nampanya F, Patel S, Pessaint L, van Ry A, Blade K, Yalley-Ogunro J, Cabus M, Brown R, Cook A, Teow E, Andersen H, Lewis MG, Lauffenburger DA, Alter G, Barouch DH, Correlates of protection against SARS-CoV-2 in rhesus macaques. Nature 590, 630–634 (2021). 33276369 10.1038/s41586-020-03041-6 PMC7906955

[10] Sabino EC, Buss LF, Carvalho MPS, Prete CA Jr., Crispim MAE, Fraiji NA, Pereira RHM, Parag KV, da Silva Peixoto P, Kraemer MUG, Oikawa MK, Salomon T, Cucunuba ZM, Castro MC, de Souza Santos AA, Nascimento VH, Pereira HS, Ferguson NM, Pybus OG, Kucharski A, Busch MP, Dye C, Faria NR, Resurgence of COVID-19 in Manaus, Brazil, despite high seroprevalence. Lancet 397, 452–455 (2021). 33515491 10.1016/S0140-6736(21)00183-5 PMC7906746

[11] Buss LF, Prete CA Jr., Abrahim CMM, Mendrone A Jr., Salomon T, de Almeida-Neto C, França RFO, Belotti MC, Carvalho MPSS, Costa AG, Crispim MAE, Ferreira SC, Fraiji NA, Gurzenda S, Whittaker C, Kamaura LT, Takecian PL, da Silva Peixoto P, Oikawa MK, Nishiya AS, Rocha V, Salles NA, de Souza Santos AA, da Silva MA, Custer B, Parag KV, Barral-Netto M, Kraemer MUG, Pereira RHM, Pybus OG, Busch MP, Castro MC, Dye C, Nascimento VH, Faria NR, Sabino EC, Three-quarters attack rate of SARS-CoV-2 in the Brazilian Amazon during a largely unmitigated epidemic. Science 371, 288–292 (2021). 33293339 10.1126/science.abe9728 PMC7857406

[12] Wolfel R, Corman VM, Guggemos W, Seilmaier M, Zange S, Müller MA, Niemeyer D, Jones TC, Vollmar P, Rothe C, Hoelscher M, Bleicker T, Brünink S, Schneider J, Ehmann R, Zwirglmaier K, Drosten C, Wendtner C, Virological assessment of hospitalized patients with COVID-2019. Nature 581, 465–469 (2020). 32235945 10.1038/s41586-020-2196-x

[13] Dagotto G, Mercado NB, Martinez DR, Hou YJ, Nkolola JP, Carnahan RH, Crowe JE, Baric RS, Barouch DH, Comparison of subgenomic and total RNA in SARS-CoV-2-challenged rhesus macaques. J. Virol 95, (2021). 10.1128/JVI.02370-20 PMC8103707 33472939

[14] Yu J, Li Z, He X, Gebre MS, Bondzie EA, Wan H, Jacob-Dolan C, Martinez DR, Nkolola JP, Baric RS, Barouch DH, Deletion of the SARS-CoV-2 spike cytoplasmic tail increases infectivity in pseudovirus neutralization assays. J. Virol 95, e00044–21 (2021). 10.1128/JVI.00044-21 PMC8139703 33727331

[15] Mercado NB, Zahn R, Wegmann F, Loos C, Chandrashekar A, Yu J, Liu J, Peter L, McMahan K, Tostanoski LH, He X, Martinez DR, Rutten L, Bos R, van Manen D, Vellinga J, Custers J, Langedijk JP, Kwaks T, Bakkers MJG, Zuijdgeest D, Rosendahl Huber SK, Atyeo C, Fischinger S, Burke JS, Feldman J, Hauser BM, Caradonna TM, Bondzie EA, Dagotto G, Gebre MS, Hoffman E, Jacob-Dolan C, Kirilova M, Li Z, Lin Z, Mahrokhian SH, Maxfield LF, Nampanya F, Nityanandam R, Nkolola JP, Patel S, Ventura JD, Verrington K, Wan H, Pessaint L, van Ry A, Blade K, Strasbaugh A, Cabus M, Brown R, Cook A, Zouantchangadou S, Teow E, Andersen H, Lewis MG, Cai Y, Chen B, Schmidt AG, Reeves RK, Baric RS, Lauffenburger DA, Alter G, Stoffels P, Mammen M, van Hoof J, Schuitemaker H, Barouch DH, Single-shot Ad26 vaccine protects against SARS-CoV-2 in rhesus macaques. Nature 586, 583–588 (2020). 32731257 10.1038/s41586-020-2607-z PMC7581548

[16] Yu J, Tostanoski LH, Peter L, Mercado NB, McMahan K, Mahrokhian SH, Nkolola JP, Liu J, Li Z, Chandrashekar A, Martinez DR, Loos C, Atyeo C, Fischinger S, Burke JS, Slein MD, Chen Y, Zuiani A, Lelis FJN, Travers M, Habibi S, Pessaint L, van Ry A, Blade K, Brown R, Cook A, Finneyfrock B, Dodson A, Teow E, Velasco J, Zahn R, Wegmann F, Bondzie EA, Dagotto G, Gebre MS, He X, Jacob-Dolan C, Kirilova M, Kordana N, Lin Z, Maxfield LF, Nampanya F, Nityanandam R, Ventura JD, Wan H, Cai Y, Chen B, Schmidt AG, Wesemann DR, Baric RS, Alter G, Andersen H, Lewis MG, Barouch DH, DNA vaccine protection against SARS-CoV-2 in rhesus macaques. Science 369, 806–811 (2020). 32434945 10.1126/science.abc6284 PMC7243363

[17] Jacob-Dolan C, Feldman J, McMahan K, Yu J, Zahn R, Wegmann F, Schuitemaker H, Schmidt AG, Barouch DH, Coronavirus-specific antibody cross reactivity in rhesus macaques following SARS-CoV-2 vaccination and infection. J. Virol 95, (2021). 10.1128/JVI.00117-21 PMC8139699 33692201

[18] Deng W, Bao L, Liu J, Xiao C, Liu J, Xue J, Lv Q, Qi F, Gao H, Yu P, Xu Y, Qu Y, Li F, Xiang Z, Yu H, Gong S, Liu M, Wang G, Wang S, Song Z, Liu Y, Zhao W, Han Y, Zhao L, Liu X, Wei Q, Qin C, Primary exposure to SARS-CoV-2 protects against reinfection in rhesus macaques. Science 369, 818–823 (2020). 32616673 10.1126/science.abc5343 PMC7402625

[19] Tostanoski L, Yu J, Mercado NB, Mahan KM, Jacob-Dolan C, Martinot AJ, Piedra-Mora C, Anioke T, Chang A, Giffin VM, Hope DL, Wan H, Bondzie EA, Mahrokhian SH, Wrijil LM, Bauer K, Pessaint L, Porto M, Piegols J, Faudree A, Kar BS, Amanat F, Krammer F, Andersen H, Lewis MG, Wegmann F, Zahn R, Schuitemaker H, Barouch DH, Immunity elicited by natural infection or Ad26.COV2.S vaccination protects hamsters against SARS-CoV-2 variants of concern. Sci. Transl. Med 13, eabj3789 (2021). 34705477 10.1126/scitranslmed.abj3789 PMC8818312

[20] Rydyznski Moderbacher C, Ramirez SI, Dan JM, Grifoni A, Hastie KM, Weiskopf D, Belanger S, Abbott RK, Kim C, Choi J, Kato Y, Crotty EG, Kim C, Rawlings SA, Mateus J, Tse LPV, Frazier A, Baric R, Peters B, Greenbaum J, Saphire EO, Smith DM, Sette A, Crotty S, Antigen-specific adaptive immunity to SARS-CoV-2 in acute COVID-19 and associations with age and disease severity. Cell 183, 996–1012.e19 (2020). 33010815 10.1016/j.cell.2020.09.038 PMC7494270

[21] Dan JM, Mateus J, Kato Y, Hastie KM, Yu ED, Faliti CE, Grifoni A, Ramirez SI, Haupt S, Frazier A, Nakao C, Rayaprolu V, Rawlings SA, Peters B, Krammer F, Simon V, Saphire EO, Smith DM, Weiskopf D, Sette A, Crotty S, Immunological memory to SARS-CoV-2 assessed for up to 8 months after infection. Science 371, (2021). 10.1126/science.abf4063 PMC7919858 33408181

[22] Grifoni A, Weiskopf D, Ramirez SI, Mateus J, Dan JM, Moderbacher CR, Rawlings SA, Sutherland A, Premkumar L, Jadi RS, Marrama D, de Silva AM, Frazier A, Carlin AF, Greenbaum JA, Peters B, Krammer F, Smith DM, Crotty S, Sette A, Targets of T cell responses to SARS-CoV-2 coronavirus in humans with COVID-19 disease and unexposed individuals. Cell 181, 1489–1501.e15 (2020). 32473127 10.1016/j.cell.2020.05.015 PMC7237901

[23] Vidal SJ, Collier ARY, Yu J, McMahan K, Tostanoski LH, Ventura JD, Aid M, Peter L, Jacob-Dolan C, Anioke T, Chang A, Wan H, Aguayo R, Ngo D, Gerszten RE, Seaman MS, Barouch DH, Correlates of neutralization against SARS-CoV-2 variants of concern by early pandemic sera. J. Virol 95, e0040421 (2021). 33893169 10.1128/JVI.00404-21 PMC8223959

