# Supplementary material for: Prior infection with SARS-CoV-2 WA1/2020 partially protects rhesus macaques against reinfection with B.1.1.7 and B.1.351 variants
Source: Sci Transl Med. 2021 Sep 21;13(618):eabj2641. doi: 10.1126/scitranslmed.abj2641 (PMC8829873; doi:10.1126/scitranslmed.abj2641)
Supplement: Supplementary file 1 — Figs. S1 to S11 [file scitranslmed.abj2641_sm.pdf]

Science Translational Medicine
AAAS

# Supplementary Materials for

## Prior infection with SARS-CoV-2 WA1/2020 partially protects rhesus macaques against reinfection with B.1.1.7 and B.1.351 variants

Abishek Chandrashekar *et al*.

Corresponding author: Dan H. Barouch, dbarouch@bidmc. harvard.edu

*Sci. Transl. Med.* **13**, eabj2641 (2021)
DOI: 10.1126/scitranslmed.abj2641

**The PDF file includes:**

Figs. S1 to S11

**Other Supplementary Material for this manuscript includes the following:**

Data file S1

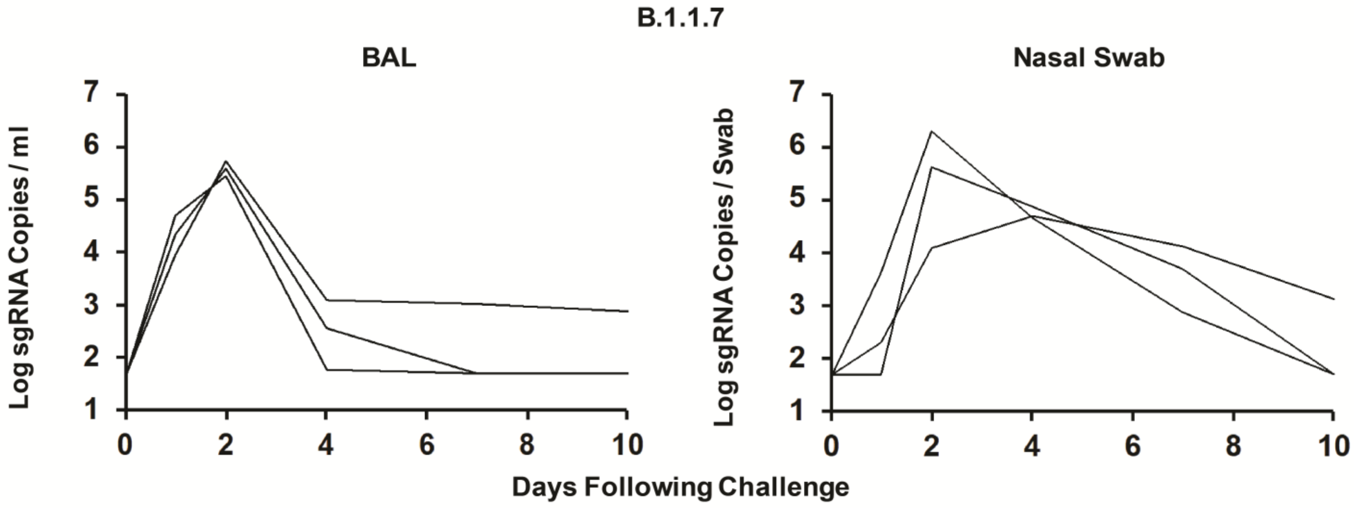


**Figure S1. SARS-CoV-2 B.1.1.7 infection of rhesus macaques.** Rhesus macaques were infected by the intranasal and intratracheal routes with $5x10^5$ median tissue culture infectious dose ($TCID_{50}$) severe acute respiratory syndrome coronavirus 2 (SARS-CoV-2) strain B.1.1.7 (n=3). Peak $log_{10}$ subgenomic RNA (sgRNA) copies/ml (limit of quantification 50 copies/ml) were assessed in bronchoalveolar lavage (BAL) following challenge, and peak $log_{10}$ sgRNA copies/swab (limit of quantification 50 copies/swab) were assessed in nasal swabs following challenge.

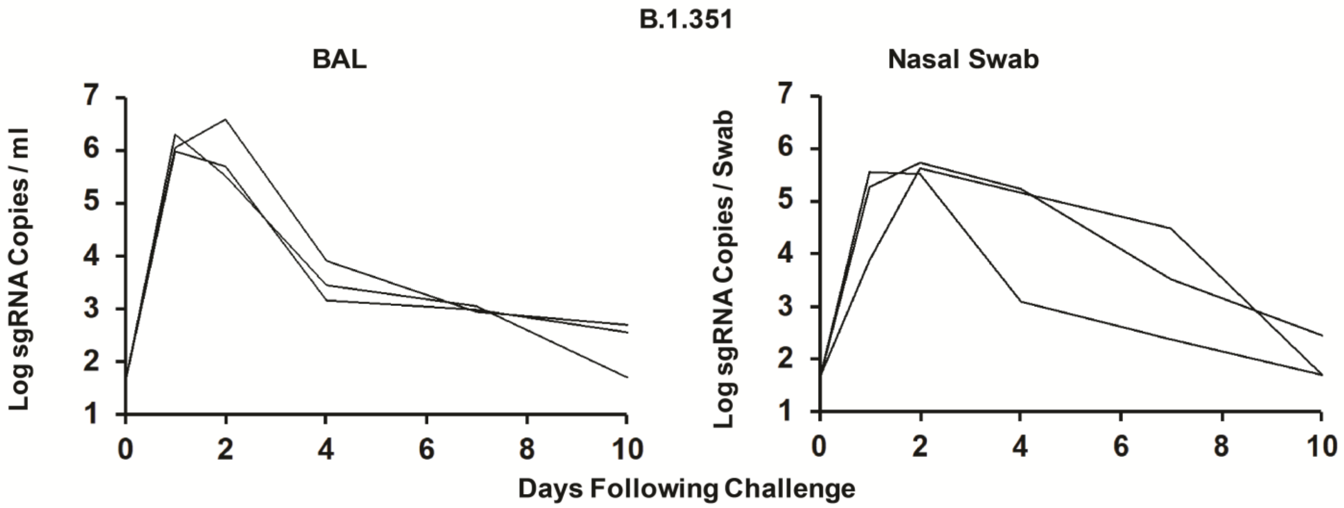


**Figure S2. SARS-CoV-2 B.1.351 infection of rhesus macaques.** Rhesus macaques were infected by the intranasal and intratracheal routes with $5x10^5$ $TCID_{50}$ SARS-CoV-2 B.1.351 (n=3). Peak $log_{10}$ sgRNA copies/ml (limit of quantification 50 copies/ml) were assessed in bronchoalveolar lavage (BAL) following challenge, and peak $log_{10}$ sgRNA copies/swab (limit of quantification 50 copies/swab) were assessed in nasal swabs following challenge.

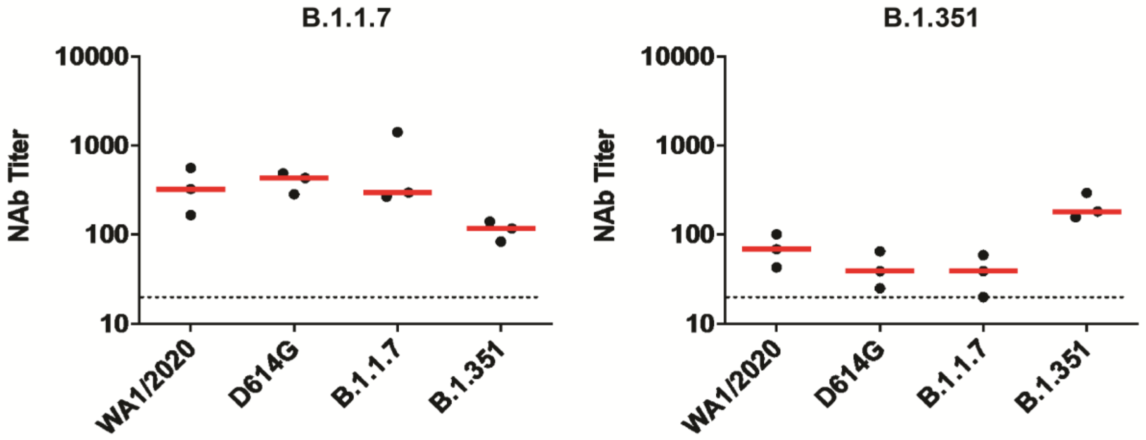


**Figure S3. Neutralizing antibody responses in B.1.1.7 and B.1.351 infected rhesus macaques.** Pseudovirus neutralizing antibody (NAb) assays against the SARS-CoV-2 WA1/2020, D614G, B.1.1.7, and B.1.351 variants were assessed in macaques at week 4 following infection with SARS-CoV-2 B.1.1.7 and B.1.351 (**fig. S1 and S2**). Horizontal red bars reflect median responses. Dotted lines reflect assay limit of quantitation.

| Day 0 (IN+IT) | Day 35 (IN+IT) | N |
|---|---|---|
| WA1/2020 | WA1/2020 | 6 |
| WA1/2020 | B.1.1.7 | 6 |
| WA1/2020 | B.1.351 | 6 |
| Sham | B.1.351 | 6* |

*3 concurrent, 3 from pilot study

**Figure S4. Study schema for re-challenge study.** IN, intranasal; IT, intratracheal.

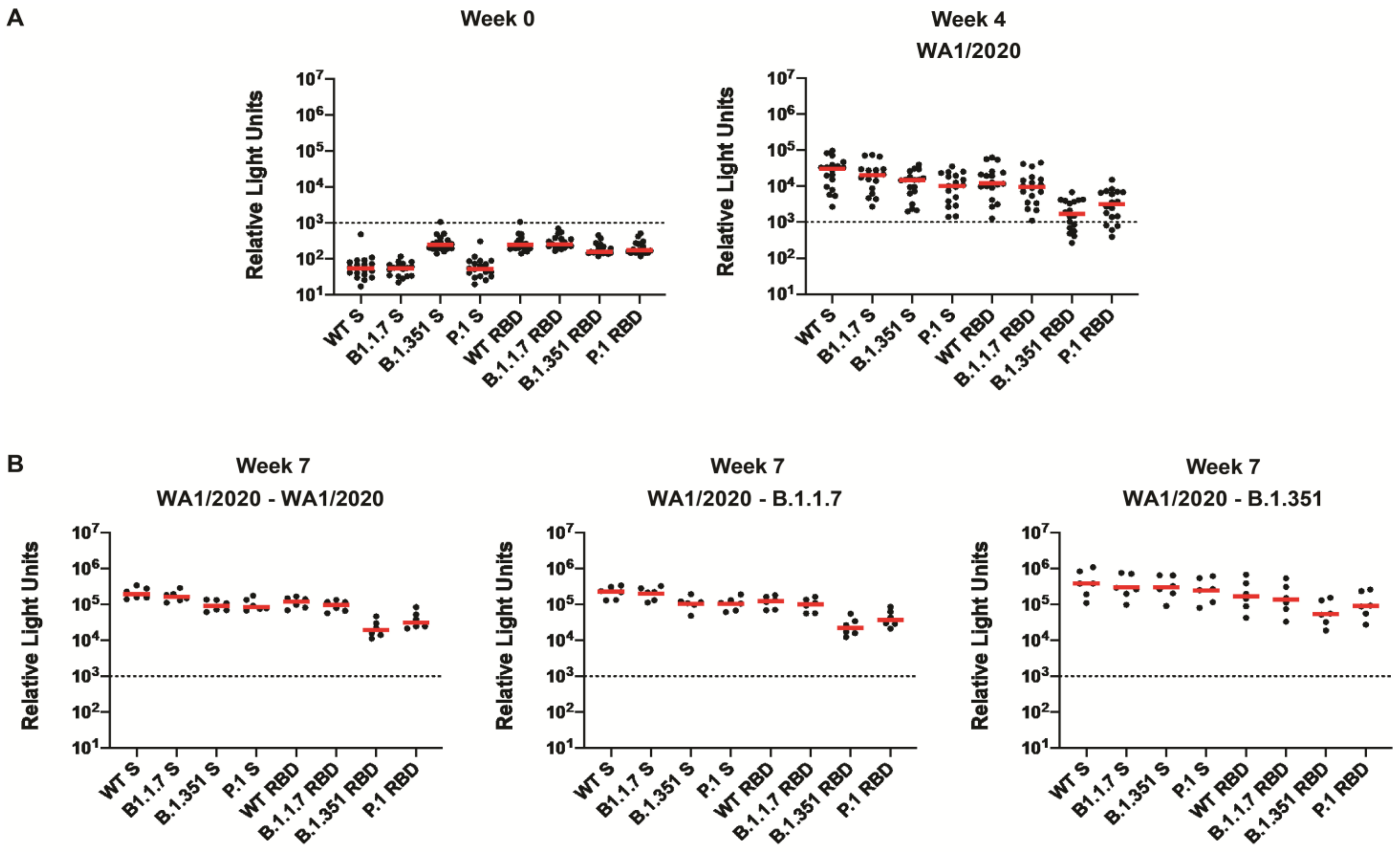


**Figure S5. Binding antibody responses in rhesus macaques by electrochemiluminescence assay (ECLA).** Spike protein (S)- and receptor binding domain (RBD)-specific binding antibody responses against the SARS-CoV-2 WA1/2020, B.1.1.7, B.1.351, and P.1 variants were assessed by ECLA (**A**) at week 0 and week 4 following primary WA1/2020 infection and (**B**) at week 7 following re-challenge with WA1/2020, B.1.1.7, and B.1.351. Horizontal red bars reflect median responses. n=6 per group. Dotted lines reflect assay limit of quantitation.

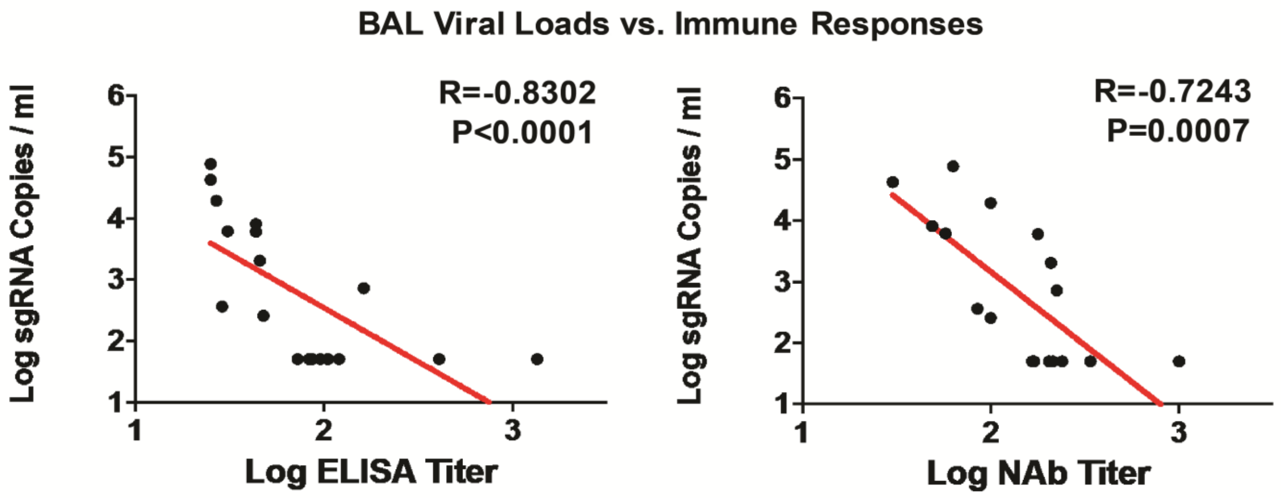


**Figure S6. Correlates of protection in BAL.** Correlations of log peak sgRNA copies/ml in BAL following re-challenge versus log enzyme-linked immunosorbent assay (ELISA) titers and log NAb titers against the re-challenge virus (WA1/2020, B.1.1.7, or B.1.351) prior to re-challenge. Red lines reflect the best linear fit relationship between these variables. P and R values reflect two-sided Spearman rank-correlation tests.

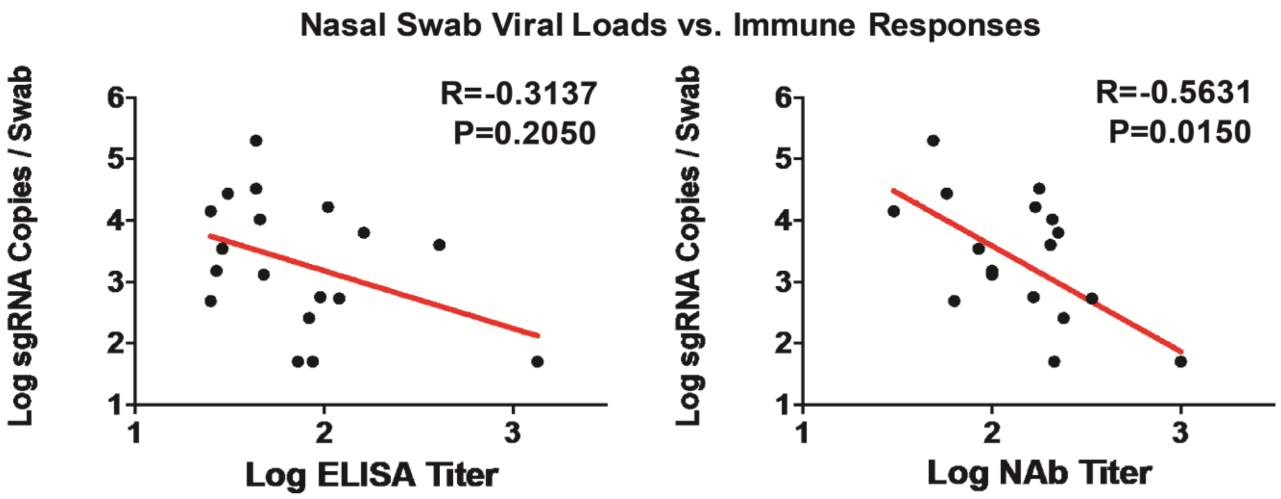


**Figure S7. Correlates of protection in nasal swabs.** Correlations of log peak sgRNA copies/swab in nasal swabs following re-challenge versus log ELISA titers and log NAb titers against the re-challenge virus (WA1/2020, B.1.1.7, or B.1.351) prior to re-challenge. Red lines reflect the best linear fit relationship between these variables. P and R values reflect two-sided Spearman rank-correlation tests.

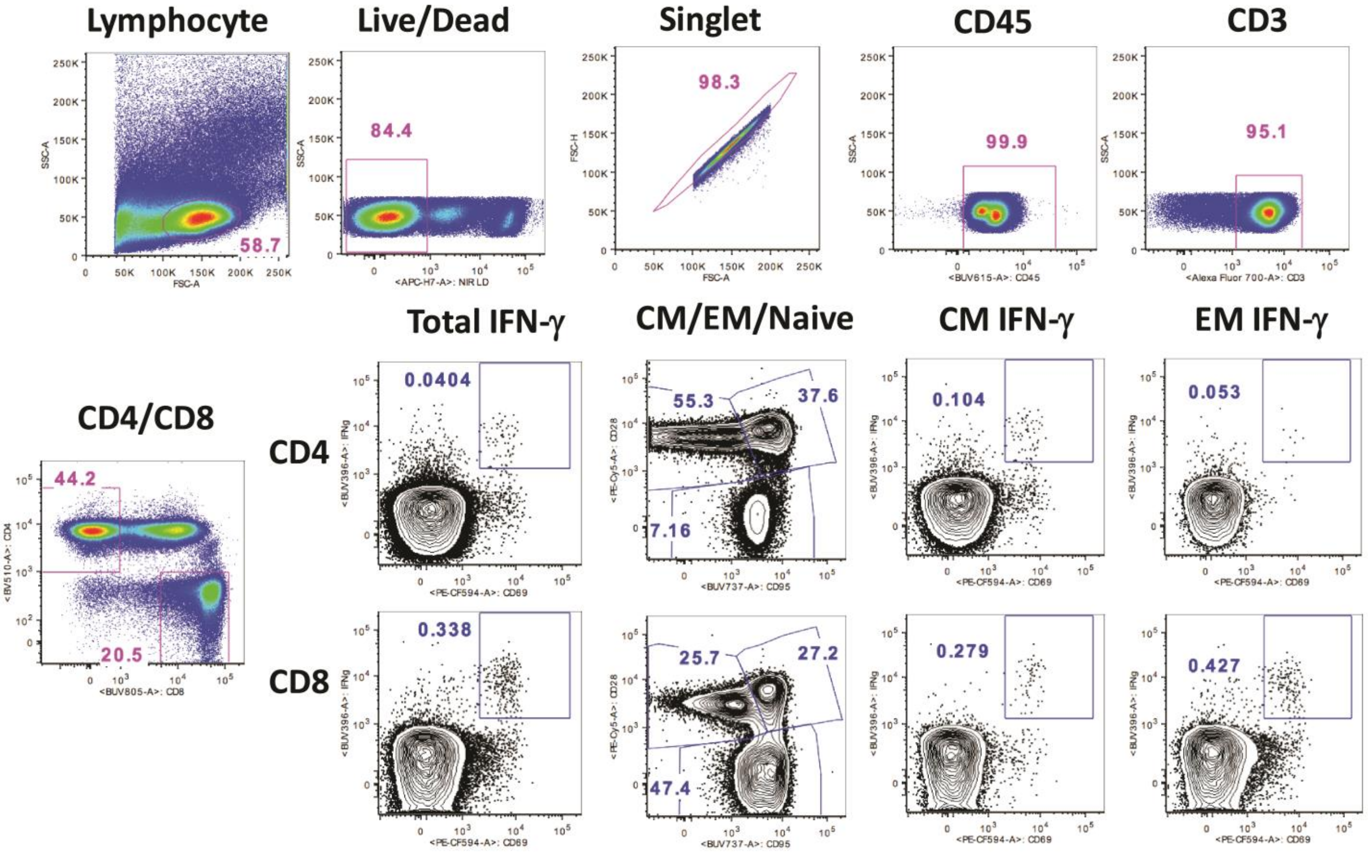


**Figure S8. Flow cytometry gating strategy for intracellular cytokine staining assays.** IFN, interferon; CM, central memory; EM, effector memory.

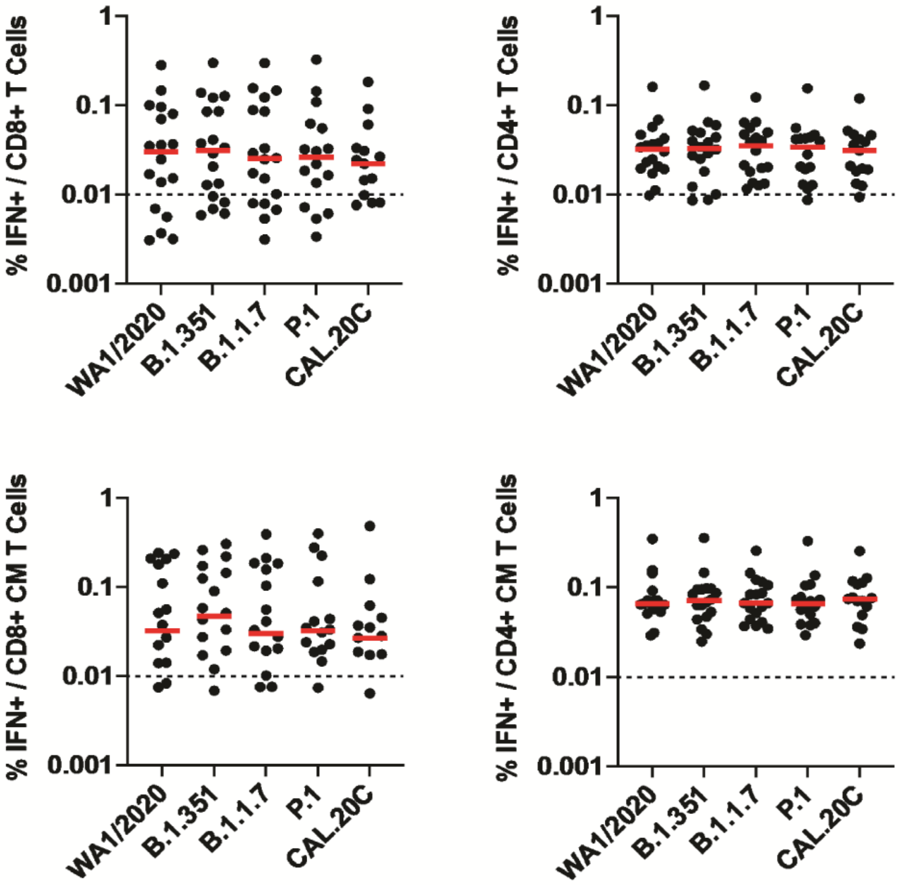


**Figure S9. T cell responses in vaccinated rhesus macaques.** Cellular immune responses to pooled spike protein peptides were assessed by IFN-γ intracellular cytokine staining (ICS) assays at week 7 to WA1/2020, B.1.351, B.1.1.7, P.1, and CAL.20C variants. ICS assays show responses of total and $CD28^{+}CD95^{+}$ central memory (CM; $T_{CM}$) $CD8^{+}$ and $CD4^{+}$ T cells. Horizontal red bars reflect median responses. Dotted lines reflect assay limit of quantitation.

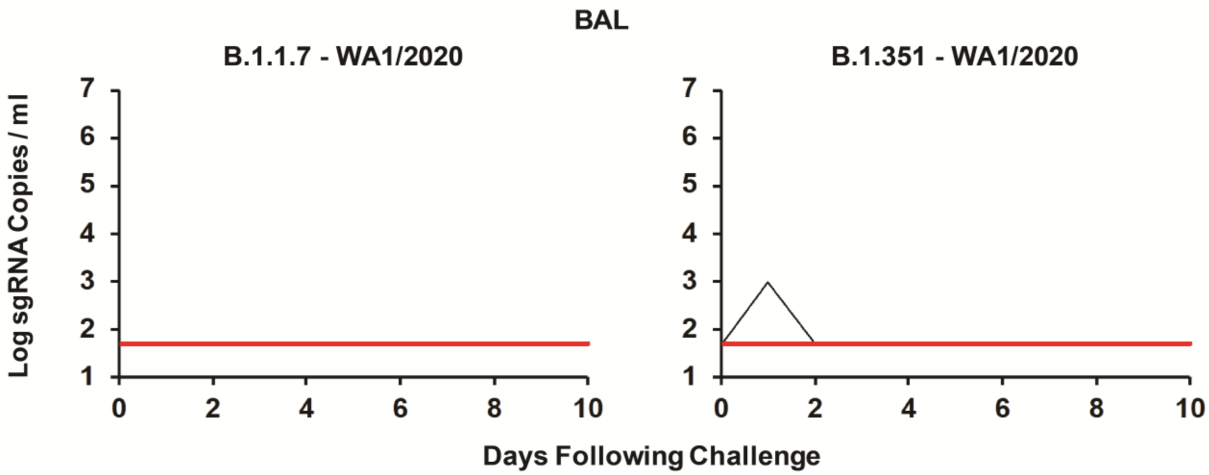


**Figure S10. Protective efficacy against re-challenge of SARS-CoV-2 B.1.1.7 and B.1.351 infected macaques with the WA1/2020 strain in BAL.** SARS-CoV-2 B.1.1.7 and B.1.351 infected rhesus macaques (**fig. S1 to S3**) were re-challenged on day 35 by the intranasal and intratracheal routes with $5x10^5$ $TCID_{50}$ SARS-CoV-2 WA1/2020 (n=3 per group). Peak $log_{10}$ sgRNA copies/ml (limit of quantification 50 copies/ml) were assessed in bronchoalveolar lavage (BAL) following challenge. Red lines reflect median values.

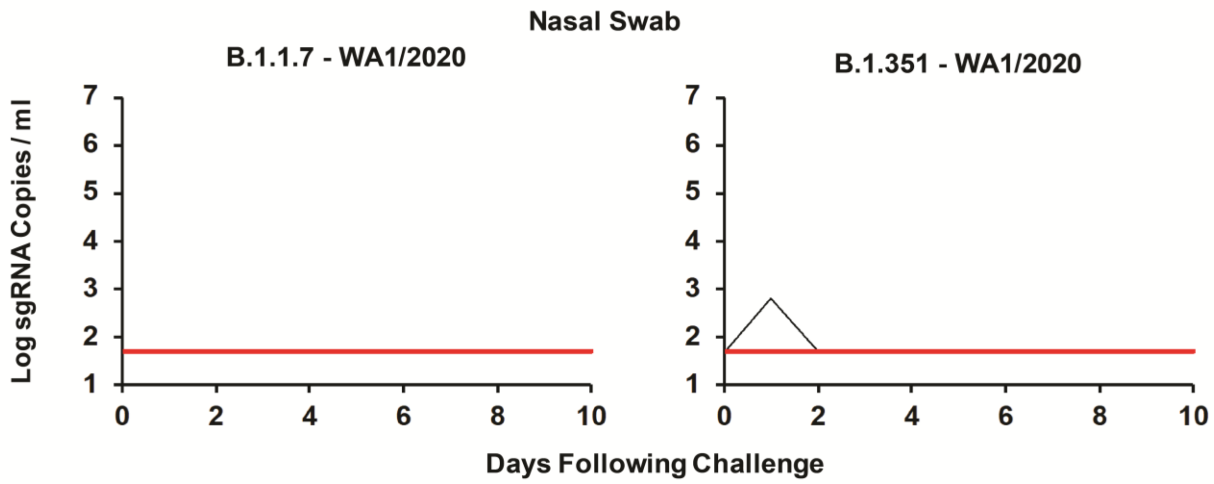


**Figure S11. Protective efficacy against re-challenge of SARS-CoV-2 B.1.1.7 and B.1.351 infected macaques with the WA1/2020 strain in nasal swabs.** In the experiment shown in **fig. S10**, peak $log_{10}$ sgRNA copies/ml (limit of quantification 50 copies/ml) were assessed in nasal swabs following challenge. Red lines reflect median values.
