# Supplementary material for: Prior infection with SARS-CoV-2 WA1/2020 partially protects rhesus macaques against reinfection with B.1.1.7 and B.1.351 variants
Source: Sci Transl Med. Author manuscript; Available in PMC 2022 Feb 10. (PMC8829873; doi:10.1126/scitranslmed.abj2641)
Supplement: Supplementary file 1 [file NIHMS1757749-supplement-Supplemental_Figures.docx]

**Figure S1.  SARS-CoV-2 B.1.1.7 infection of rhesus macaques.** Rhesus macaques were infected by the intranasal and intratracheal routes with 5x10^5^ median tissue culture infectious dose (TCID_50_) severe acute respiratory syndrome coronavirus 2 (SARS-CoV-2) strain B.1.1.7 (n=3).  Peak log_10_ subgenomic RNA (sgRNA) copies/ml (limit of quantification 50 copies/ml) were assessed in bronchoalveolar lavage (BAL) following challenge, and peak log_10_ sgRNA copies/swab (limit of quantification 50 copies/swab) were assessed in nasal swabs following challenge.

**Figure S2.  SARS-CoV-2 B.1.351 infection of rhesus macaques.** Rhesus macaques were infected by the intranasal and intratracheal routes with 5x10^5^ TCID_50_ SARS-CoV-2 B.1.351 (n=3). Peak log_10_ sgRNA copies/ml (limit of quantification 50 copies/ml) were assessed in bronchoalveolar lavage (BAL) following challenge, and peak log_10_ sgRNA copies/swab (limit of quantification 50 copies/swab) were assessed in nasal swabs following challenge.

**Figure S3.  Neutralizing antibody responses in B.1.1.7 and B.1.351 infected rhesus macaques.**  Pseudovirus neutralizing antibody (NAb) assays against the SARS-CoV-2 WA1/2020, D614G, B.1.1.7, and B.1.351 variants were assessed in macaques at week 4 following infection with SARS-CoV-2 B.1.1.7 and B.1.351 (**fig. S1 and S2**).  Horizontal red bars reflect median responses.  Dotted lines reflect assay limit of quantitation.

**Figure S4. Study schema for re-challenge study.** IN, intranasal; IT, intratracheal.

**Figure S5.  Binding antibody responses in rhesus macaques by electrochemiluminescence assay (ECLA).**  Spike protein (S)- and receptor binding domain (RBD)-specific binding antibody responses against the SARS-CoV-2 WA1/2020, B.1.1.7, B.1.351, and P.1 variants were assessed by ECLA (**A**) at week 0 and week 4 following primary WA1/2020 infection and (**B**) at week 7 following re-challenge with WA1/2020, B.1.1.7, and B.1.351.  Horizontal red bars reflect median responses. n=6 per group.  Dotted lines reflect assay limit of quantitation.

**Figure S6.  Correlates of protection in BAL.**  Correlations of log peak sgRNA copies/ml in BAL following re-challenge versus log enzyme-linked immunosorbent assay (ELISA) titers and log NAb titers against the re-challenge virus (WA1/2020, B.1.1.7, or B.1.351) prior to re-challenge.  Red lines reflect the best linear fit relationship between these variables.  P and R values reflect two-sided Spearman rank-correlation tests.

**Figure S7.  Correlates of protection in nasal swabs.**  Correlations of log peak sgRNA copies/swab in nasal swabs following re-challenge versus log ELISA titers and log NAb titers against the re-challenge virus (WA1/2020, B.1.1.7, or B.1.351) prior to re-challenge.  Red lines reflect the best linear fit relationship between these variables.  P and R values reflect two-sided Spearman rank-correlation tests.

**Figure S8.  Flow cytometry gating strategy for intracellular cytokine staining assays.** IFN, interferon; CM, central memory; EM, effector memory. 

**Figure S9.  T cell responses in vaccinated rhesus macaques.**  Cellular immune responses to pooled spike protein peptides were assessed by IFN-γ intracellular cytokine staining (ICS) assays at week 7 to WA1/2020, B.1.351, B.1.1.7, P.1, and CAL.20C variants.  ICS assays show responses of total and CD28^+^CD95^+^ central memory (CM; T_CM_) CD8^+^ and CD4^+^ T cells. Horizontal red bars reflect median responses. Dotted lines reflect assay limit of quantitation.

**Figure S10.  Protective efficacy against re-challenge of SARS-CoV-2 B.1.1.7 and B.1.351 infected macaques with the WA1/2020 strain in BAL.**  SARS-CoV-2 B.1.1.7 and B.1.351 infected rhesus macaques (**fig. S1 to S3**) were re-challenged on day 35 by the intranasal and intratracheal routes with 5x10^5^ TCID_50_ SARS-CoV-2 WA1/2020 (n=3 per group).  Peak log_10_ sgRNA copies/ml (limit of quantification 50 copies/ml) were assessed in bronchoalveolar lavage (BAL) following challenge.  Red lines reflect median values.

**Figure S11.  Protective efficacy against re-challenge of SARS-CoV-2 B.1.1.7 and B.1.351 infected macaques with the WA1/2020 strain in nasal swabs.**  In the experiment shown in **fig. S10**, peak log_10_ sgRNA copies/ml (limit of quantification 50 copies/ml) were assessed in nasal swabs following challenge.  Red lines reflect median values.
